# Giant proximal pulmonary pseudoaneurysm treated with stenting and Cyanoacrylate glue: a technical case report

**DOI:** 10.1186/s42155-024-00461-7

**Published:** 2026-04-24

**Authors:** Nicolas Stacoffe, Théo Bourdeyroux, Farouk Tradi, Romain L’Huillier

**Affiliations:** 1 Department of Diagnostic and Interventional Radiology, Centre Hospitalier Lyon Sud, University of Lyon, 69495 Hospices Civils de LyonPierre-Bénite, France; 2 Department of Interventional Radiology, Marseille Public University Hospital System, La Timone University Hospital, Service de radiologie - Hôpital Timone, 264 Rue Saint Pierre, 13385 Marseille CEDEX 05, France; 3 Department of Diagnostic and Interventional Radiology, Hôpital Edouard Herriot, University of Lyon, 69003 Hospices Civils de LyonLyon, France; 4 LabTAU - INSERM U1032, 69003 Lyon, France; 5 The French Comprehensive Liver Center, Hospices Civils de Lyon, University of Lyon, 69004 Lyon, France

**Keywords:** Pseudo aneurism, Embolization, Stenting, Glue, Pulmonary artery

## Abstract

Pulmonary artery pseudoaneurysms pose a therapeutic challenge due to their lack of a defined wall, making the choice of endovascular therapy crucial. The added complexity arises from their proximal location, often necessitating endovascular stenting for effective treatment. Our case highlights a successful therapeutic intervention involving stenting and glue embolization to address a sizable proximal pseudoaneurysm in the right pulmonary artery. This approach demonstrates the efficacy of combining these techniques in managing such challenging cases. The utilization of endovascular stenting, coupled with glue embolization, could be a therapeutic option in the context of giant pseudoaneurysms located proximally within the pulmonary artery.

## Introduction

Hemoptysis is a medical emergency commonly encountered in interventional radiology, often requiring embolization [1]. The origin of this bleeding is primarily tumoral, infectious, or related to bronchial dilation. In the majority of cases (>90%), the bleeding originates from the systemic bronchial system, necessitating embolization of bronchial arteries [2]. In certain cases, especially in tumoral or infectious scenarios, the bleeding may arise from the pulmonary arteries and requires rapid treatment because of the risk of rupture associated with a high mortality rate [1]. Embolization is performed by accessing the right cardiac cavities after venous system puncture [1]. When the pseudoaneurysm is proximal to the pulmonary artery, managing these pseudoaneurysms becomes even more complex and poses a therapeutic challenge [3]. This case describes the treatment of a giant pseudoaneurysm involving the right pulmonary artery.

## Case Presentation

**Fig. 1:**
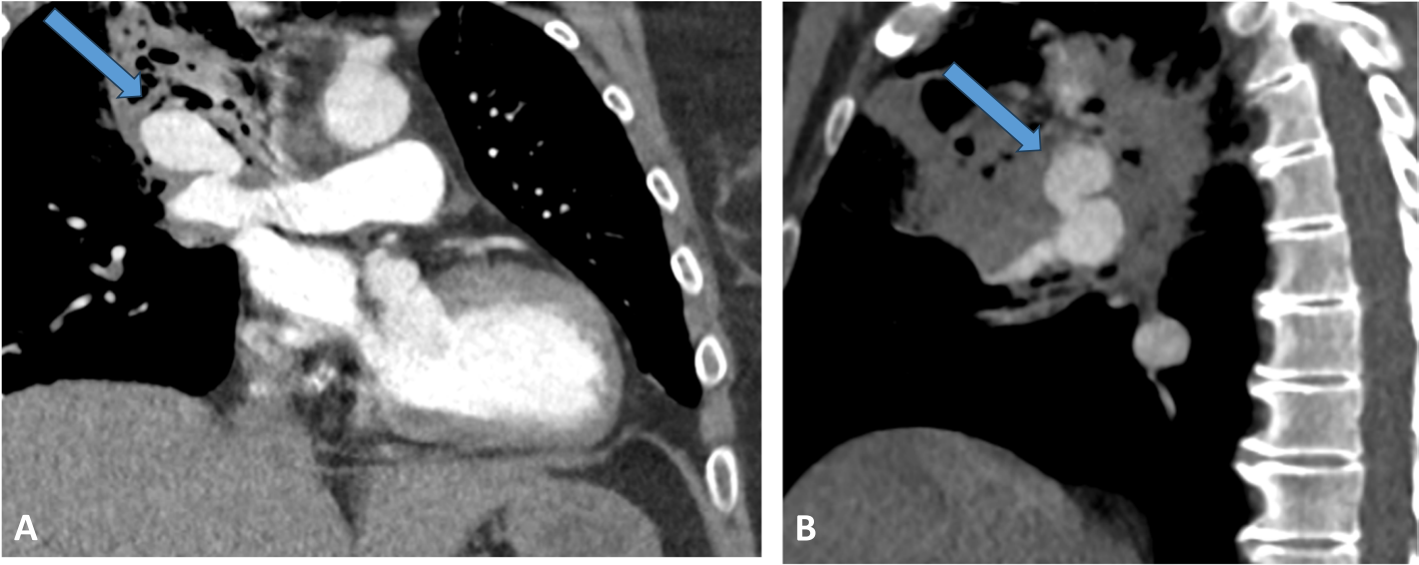
CT scan revealing a proximal false aneurysm of the right pulmonary artery (blue arrow) in coronal section **A** and sagittal section **B**

**Fig. 2:**
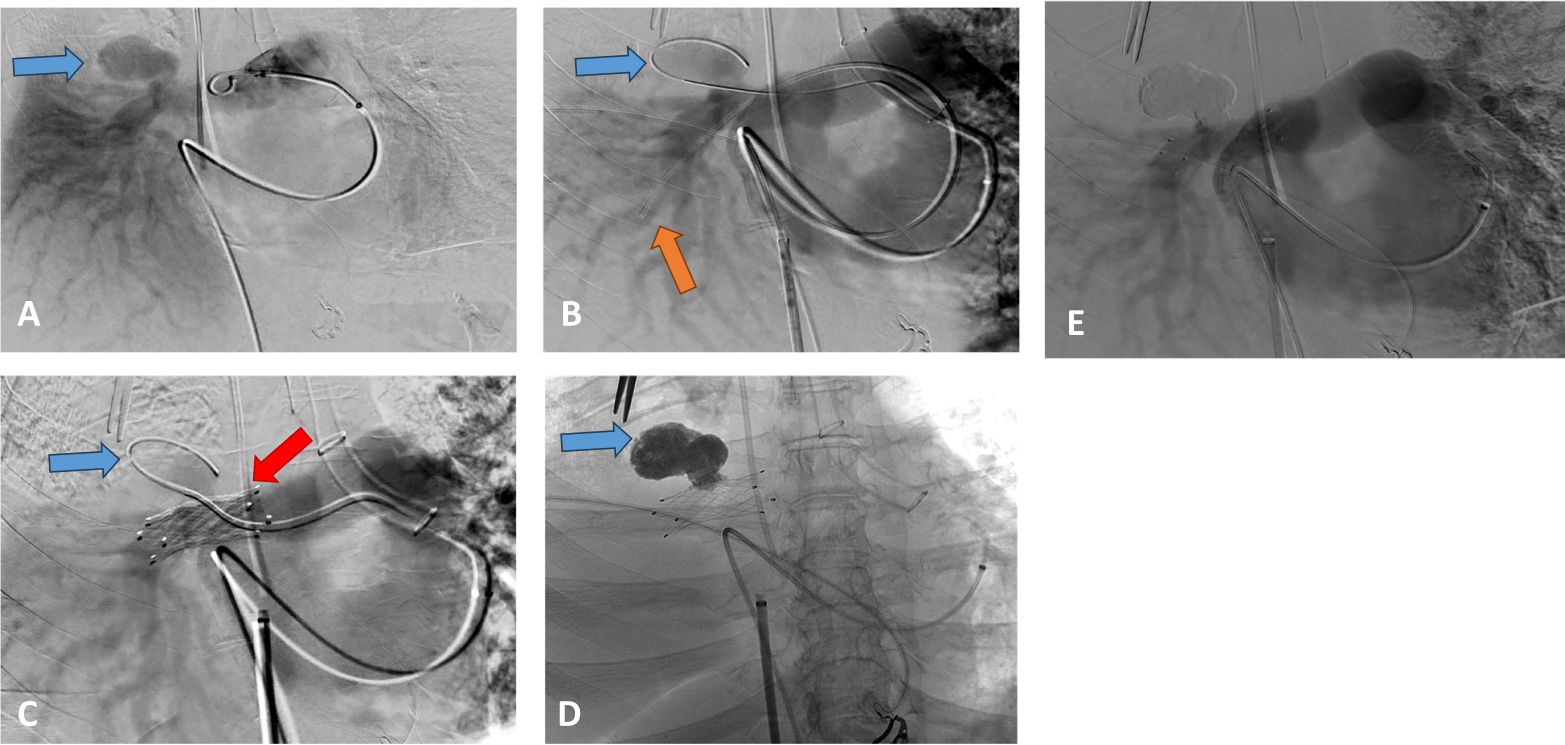
Various stages of pseudoaneurysm treatment. **A** Angiography within the right pulmonary artery revealing a giant pseudoaneurysm approximately 3 cm in size (blue arrow).**B** Microcatheter left within the false aneurysm (blue arrow), with a vertebral catheter positioned in a lower segmental artery (orange arrow). **C** Stent in place (red arrow) and a patent but stagnant aneurysm with the microcatheter still in position (blue arrow). **D** Final result after injecting approximately 5 cc of glue. **E** Concluding angiography depicting a patent pulmonary artery with complete exclusion of the pseudoaneurysm

We present the case of a 54-year-old female who presented with massive hemoptysis (approximately 400 mL), treated for a tumor in the right upper lobe of the lung with chemotherapy and radiotherapy. This patient had an apical cavity on previous scans with local-regional invasion. She had already undergone embolization of the right bronchial artery for hemoptysis 2 months ago. Upon arrival at the hospital, she had hemoptysis with significant desaturation to 92% on 9L of oxygen. The CT scan revealed a giant (32 mm in length with a neck measuring 9 mm) proximal pseudoaneurysm of the right pulmonary artery (Fig. 1A and B). Thoracic surgeons deemed the patient ineligible for surgery due to aggressive underlying cancer. It was decided to perform stenting while securing the pulmonary artery frome the pseudoaneurysm. The procedure was performed under general anesthesia with selective intubation in the left bronchus. We first accessed the right common femoral vein with a 6 French long sheath, reaching the right cardiac cavities and then the right pulmonary artery using a 5 French curved pigtail catheter. The initial pulmonary angiography identified the pseudoaneurysm involving the right pulmonary artery (Fig. 2A). Throughout a 5 French vertebral catheter, we delicately placed a 2.7 French micro catheter (Progreat, Terumo) within the pseudoaneurysm. Next, we accessed the left common femoral vein with a 10 French long sheath, also returning to the right cardiac cavities with a 5 French curved pigtail catheter. Subsequently, we navigated to a lower lobar artery with the vertebral catheter (Fig. 2B). Using a long rigid 260 cm Amplatz guide wire with a short flopy tip, we deployed a self-expanding nitinol-covered stent measuring 13.5 mm in diameter and 40 mm in length (Fluency, Bard). Other stent sizes were not available during the procedure.

The right pulmonary artery measured between 12.5 and 14.5 mm in the segment, indicating an expectation of an endoleak around the stent. Indeed, on the deployed stent series, there was a tiny passage of contrast within the pseudoaneurysm (Fig. 2C) . Additionally, during injection through the microcatheter, the contrast stagnated significantly. Consequently, we decided to embolize the pseudoaneurysm by injecting a liquid agent, Cyanoacrylate (3 mL) (Glubran GEM, Italy), mixed with Lipiodol (3 mL) (Guerbet, France), through the microcatheter. Considering the size of the pseudoaneurysm, we had prepared a 6 mL syringe of the mixture, which we injected slowly. The adhesive polymerized gradually around the microcatheter, and we stopped the injection when the adhesive filled the neck up to the stent (Fig. 2D). Subsequently, we performed a final angiography in the long sheath that remained in the right atrium, showing complete exclusion of the pseudoaneurysm (Fig. 2E). No antiplatelets or anticoagulation therapy was performed before or during the procedure. The patient was extubated few hours after the procedure.

**Fig. 3:**
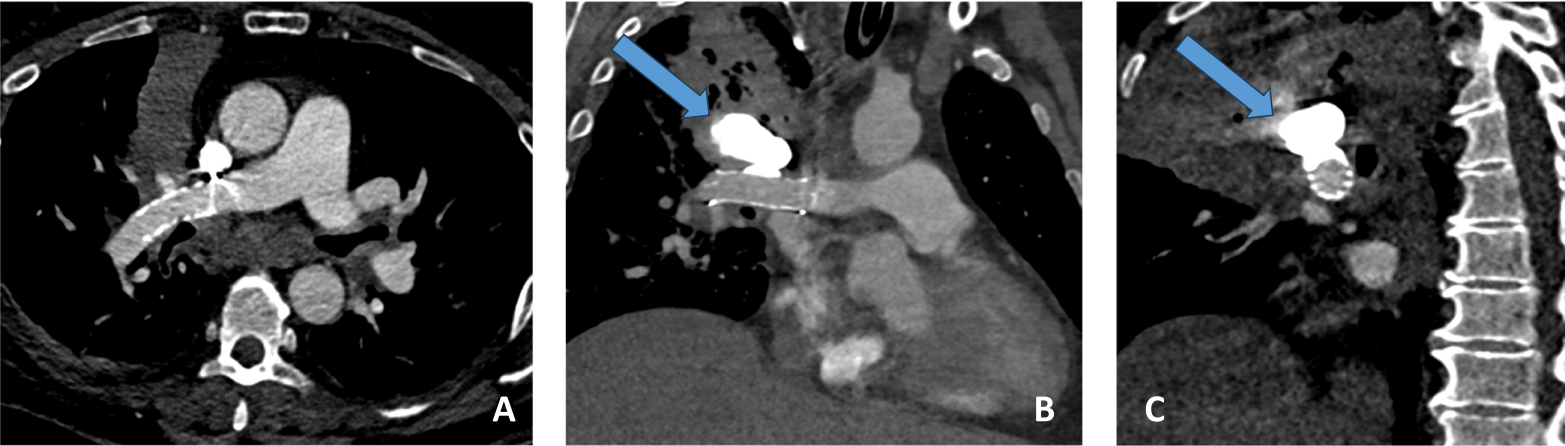
Follow-up CT scan showing a patent stent with a completely filled pseudoaneurysm using glue (blue arrow) in axial section **A**, coronal section **B**, and sagittal section **C**

**Fig. 4:**
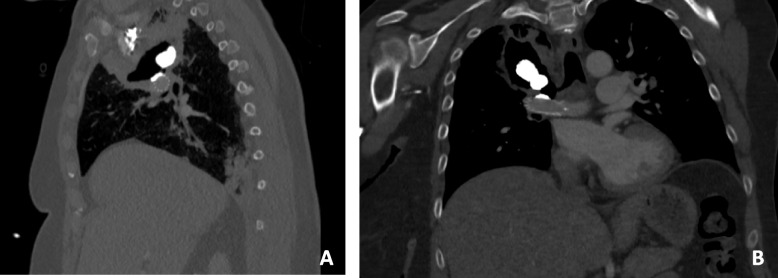
CT scan (day 10) showing a completely excluded pseudoaneurysm and no bleeding around the stent in sagittal **A** and coronal **B** section

A CT scan on Day 2 revealed a perfect exclusion of the pseudoaneurysm with a patent pulmonary artery. (Fig. 3A, B and C). No anticoagulation or antiplatelet therapy was initiated because of the risk of bleeding. On Day 3, the patient was discharged from the intensive care unit to return to the regular ward without any respiratory symptoms and chemotherapy was started. A CT scan was performed on day 10 because of recurrence of hemoptysis and still showed exclusion of pseudo aneurysm and no bleeding around stent (Fig. 4A and B). The origin of the hemoptysis was not identified and a new angiography was scheduled the following day. Unfortunately, the patient died the evening after the CT scan.

## Discussion

There is no standard management for pseudoaneurysms of the pulmonary arteries, as they remain infrequent pathologies. These pseudoaneurysms present a risk of massive hemoptysis with potential for respiratory arrest during intervention, and an untreated patient's risk of death exceeds 50% [4, 5]. Pseudoaneurysms, by definition, lack a healthy wall, increasing the risk of coil migration compared to an aneurysm with intact walls [6]. Various articles attempt to classify pseudoaneurysms based on arteriography and examinations to guide treatment decisions [7]. Surgery can be complicated or contraindicated, especially in patients with significant comorbidities such as pulmonary arterial hypertension [8]. Endovascular treatment appears to have few complications [9]. However, careful consideration of materials is necessary, as re-treatment for recurrence is required in 50% of cases [10]. Coiling is a viable option distally but involves sacrificing the carrying artery and utilizing a sandwich technique. When the pseudoaneurysm is proximal, a stent is a good option, but there is currently no material tailored to the pulmonary system. In our case, the dual approach, enabling the exclusion of a proximal pseudoaneurysm using a stent combined with a polymerizing liquid agent, seems like a favorable option, providing preservation of the main pulmonary artery trunk and definitive closure of the pseudoaneurysm. Glue was one of the many available options, but it seemed to us to be the best for completely filling the leaks around the stent. Using Onyx ® (Medtronic) would have been longer and more expensive. Coils would have been less effective for filling endoleaks. However, with a stent perfectly sized to the vessel, additional pseudoaneurysm treatment may be optional : there may be a risk of stent mobilization during exclusion. We found no consensus on the management of anticoagulant and antiplatelet regime in this situation. Because of major bleeding risk, no treatment was initiated during or after the procedure.

## Conclusion

Our case report demonstrates the possibility of a double approach with exclusion of a proximal false aneurysm by stenting and glue. It allows the false aneurysm to be secured in complete safety, while preserving flow within the pulmonary artery.

## Data Availability

Data can be obtained from the corresponding author.

## References

[1] Kalra-Lall A, Donaldson J, Martin C 3rd. Brief review: Pulmonary artery aneurysms and pseudoaneurysms. Int J Cardiovasc Imaging. 2019;35(7):1357–64. 10.1007/s10554-019-01547-3. Epub 2019 Jun 13. 31190207 10.1007/s10554-019-01547-3

[2] Crocco JA, Rooney JJ, Fankushen DS, DiBenedetto RJ, Lyons HA. Massive hemoptysis. Arch Internal Med. 1968;121(6):495–8. 5652400

[3] Pelage JP, Hajjam ME, Lagrange C, Chinet T, Vieillard-Baron A, Chagnon S, Lacombe P. Pulmonary artery interventions: an overview. RadioGraphics. 2005;25:1653–67. 16284141 10.1148/rg.256055516

[4] Jean-Baptiste E. Clinical assessment and management of massive hemoptysis. Critical Care Medicine. 2000;28(5):1642–7. 10834728 10.1097/00003246-200005000-00066

[5] Chen Y, Gilman MD, Humphrey KL, Salazar GM, Sharma A, Muniappan A, Shepard JO, Wu CC. Pulmonary artery pseudoaneurysms: clinical features and CT findings. Ame J Roentgenol. 2017;208:84–91. 10.2214/AJR.16.16312 27656954

[6] Nguyen ET, Silva CI, Seely JM, Chong S, Lee KS, Müller NL. Pulmonary artery aneurysms and pseudoaneurysms in adults: findings at CT and radiography. AJR Am J Roentgenol. 2007;188:W126–34. 17242217 10.2214/AJR.05.1652

[7] Shin S, Shin TB, Choi H, Choi JS, Kim YH, Kim CW, Jung GS, Kim Y. Peripheral pulmonary arterial pseudoaneurysms: therapeutic implications of endovascular treatment and angiographic classifications. Radiology. 2010;256(2):656–64. 20656846 10.1148/radiol.10091416

[8] McCollun WB, Mattox KL, Guinn GA, Beall AC. Immediate operative treatment for massive hemoptysis. Chest. 1975;67(2):152–5. 1116390 10.1378/chest.67.2.152

[9] Cantasdemir M, Kantarci F, Mihmanli I, Akman C, Numan F, Islak C, Bozkurt AK. Emergency endovascular management of pulmonary artery aneurysms in Behcet’s disease: report of four cases and a review of the literature. Cardiovasc Intervent Radiol. 2002;25(6):533–7. 12042999 10.1007/s00270-002-1967-0

[10] Krokidis MI, Spiliopoulos S, Ahmed IR, Gkoutzios PA, Sabharwal TA, Reidy JO. Emergency endovascular management of pulmonary artery aneurysms and pseudoaneurysms for the treatment of massive haemoptysis. Hellenic J Cardiol. 2014;55:204–10. 24862612

